# Calendar

**DOI:** 10.1155/S1463924684000407

**Published:** 1984


					M. J. Adams and G. J. Ewen Using a microcomputer with an EPR spectrometer/Calendar
Acknowledgements
The authors wish to acknowledge the assistance given by
D. McPhail in operating the spectrometer and preparing the
spectra.
References
Practical UV and Fluorescence Spectrometry
To be heldfrom 15 to 19 July 1985 in Loughborough,
UK.
Further information from Miss C. D. Newton,
Department ofChemistry, Lou#hborough University of
Technology, Loughborough, Leicestershire LE11 3TU,
UK.
1. ADAMS, M. J., MITCHELL, M. C. and EWEN, G. J., Analytica
Chimica Acta, 149 (1983), 101.
2. ADAMS, M. J. and BLACK, I. J. Journal ofAutomatic Chemistry, 5
(1983), 9.
3. EWEN, G. J. and ADAMS, M. J. (to be published).
4. WVrNE, J. Analo#ue Dialogue, 14 (1980), 9.
5. TIMtO, M. and HOLLOWAV, P., Analogue Dialogue, 12 (1978), 3.
6. EWEN, G. J. and ADAMS, M. J. (to be published).
7. McPAL, D. (unpublished work).
IUPAC 1985
To be held from 9 to 13 September 1985 in
Manchester, UK.
Further information from the Royal Society of
Chemistry, Burlington House, London W1 VOBN.
Calendar
1985 MEETINGS
36th Pittsburgh Conference and Exposition on Analyt-
ical Chemistry and Applied Spectroscopy
To be held from 25 February to March 1985 at the
Convention Center, New Orleans, Louisiana, USA.
Further informationfrom David R. Weill III, Shady Side
Academy, 423 Fox Chapel Road, Pittsburgh,
Pennsylvania 15238, USA.
Microprocessor Familiarization
Course to held from 22 to 23 April 1985 in Bromley,
UK.
Further information from Sira Ltd, South Hill,
Chislehurst, Kent BR7 5EH, UK.
Health Care--Is it Improved by the Use ofStandards in
Clinical Laboratory Science? 6th Annual General
Meeting and 6th Seminar of the European Committee
for Clinical Laboratory Standards
To be held from 8 to 10 May 1985 at Bornheim-
Walberberg, FR Germany.
Further informationfrom Irene Batty, Executive Direc-
tor ECCLS, Wellcome Research Laboratories, Langley
Court, Beckenham, Kent BR3 3BS, UK.
Moisture Measurement in Solids and Gases
Course to be held from 14 to 15 May 1985 in
Chislehurst, UK.
Further informationfrom Sira (above).
Hewlett-Packard 1985 Analytical Symposium
To be held from 11 to 13 June 1985 at the Wembly
Conference Centre, London.
Further information from Mrs Tina Meats, Hewlett-
Packard Ltd, Miller House, The Rinf4, Bracknell,
Berkshire, UK.
Colloquium Spectroscopium Internationale XXIV
To be held from 15 to 21 September 1985 in Garmisch-
Partenkirchen, FR Germany.
Further information from CSI XXIV, Organisations-
biiro, lnstitut fiir Spektrochemie und angewandte
Spektroskopie, Postfach 778, D4600 Dortmund 1, FR
Germany.
1986 MEETINGS
4th World Filtration Congress
To be held in Ostend, Belgium from 22 to 25 April
1986.
Further information from the Secretariat of World
Filtration Congress IV, KVIV-Technologisch Istituut,
Jan van Rijswicjcklaan 58, B2018 Antwerpen, Belgium.
Analytica 86
To be held from 3 to 6 June 1986 at Miinchen, FR
Germany.
Further information from Miinchener Messe- und
Ausstellungsgesellschaft mbH, Messegeliinde, Postfach
12 10 09, D8000 Miinchen 12, FR Germany.
SAC 86/3rd BNASS: an International Conference on
Analytical Chemistry
To be held from 20-26 July 1986 at the University of
Bristol, UK.
Further information from The Secretary, Analytical
Division, Royal Society ofChemistry, Burlington House,
London W1 V OBN.
Conference organizers whould sendfull details oftheir
meeting for inclusion in 'Journal of Automatic
Chemistry's' calendar at least three months before the
event. Information to the Editor, Journal ofAutomatic
Chemistry, Taylor & Francis Ltd, 4 John Street,
London WC1N 2ET.
Product Design Assurance in Engineering
To be held 11 to 13 June 1985 at the Wembley
Conference Centre, London.
Further information .from Mrs H. M. W. Gibbons,
Society of Environmental Engineers, Owles Hall,
Buntingford, Hertfordshire, UK.
205

				

